# Epidemiology of bacteremia caused by uncommon non-fermentative gram-negative bacteria

**DOI:** 10.1186/1471-2334-13-167

**Published:** 2013-04-08

**Authors:** Pinyo Rattanaumpawan, Prapassorn Ussavasodhi, Pattarachai Kiratisin, Nalinee Aswapokee

**Affiliations:** 1 Division of Infectious Diseases and Tropical Medicine, Faculty of Medicine Siriraj Hospital, Mahidol University, Bangkok, Thailand; 2 Department of Medicine, Faculty of Medicine Siriraj Hospital, Mahidol University, Bangkok, Thailand; 3 Department of Microbiology, Faculty of Medicine Siriraj Hospital, Mahidol University, Bangkok, Thailand

**Keywords:** Bacteremia, Non-fermentative gram-negative bacteria, Epidemiology, *Pseudomonas aeruginosa*, *Acinetobacter baumannii*

## Abstract

**Background:**

Prevalence of bacteremia caused by non-fermentative gram-negative bacteria (NFGNB) has been increasing over the past decade. Although many studies have already investigated epidemiology of NFGNB bacteremia, most focused only on common NFGNB including *Pseudomonas aeruginosa* (PA) and *Acinetobacter baumannii *(AB). Knowledge of uncommon NFGNB bacteremia is very limited. Our study aimed to investigate epidemiology and identify factors associated with uncommon NFGNB bacteremia.

**Methods:**

This observational study was conducted at a university hospital in Thailand during July 1, 2007-Dec 31, 2008. All patients who had at least one blood culture positive for NFGNB and met the criteria for systemic inflammatory response syndrome within 24 hours before/after obtaining the blood culture were enrolled. The NFGNB isolates that could not be satisfactorily identified by the standard biochemical assays were further characterized by molecular sequencing methods. To identify factors associated with uncommon NFGNB bacteremia, characteristics of patients in the uncommon NFGNB group were subsequently compared to patients in the common NFGNB group (AB and PA bacteremia).

**Results:**

Our study detected 223 clinical isolates of NFGNB in 221 unique patients. The major causative pathogens were *AB* (32.7%), followed by *P*A (27.8%), *Stenotrophomonas maltophilia* (5.4%), *Acinetobacter lwoffii* (4.9%) and *Burkholderia pseudomallei* (2.7%). Infection-related mortality was 63.0% in the AB group*,* 40.3% in the PA group and 17.4% in the uncommon NFGNB group. Factors associated with uncommon NFGNB bacteremia (OR [95% CI]; p-value) were male sex (0.28 [0.14-0.53]; p < 0.001), hospital-acquired infection (0.23 [0.11-0.51]; p < 0.001), recent aminoglycosides exposure 0.23 [0.06-0.8]; p = 0.01), primary bacteremia (6.43 [2.89-14.2]; p < 0.001]), catheter related infection (4.48 [1.54-13.06]; p < 0.001) and recent vancomycin exposure (3.88 [1.35-11.1]; p = 0.02).

**Conclusions:**

Our distribution of causative pathogens was slightly different from other studies. The common NFGNB group had a remarkably higher ID-mortality than the uncommon NFGNB group. Knowledge of factors associated with uncommon NFGNB bacteremia would help physicians to distinguish between low vs. high risk patients.

## Background

Bacteremia is a serious infection which is associated with high morbidity and mortality [1]. Gram-negative bacteria have been documented as the most common cause of bacteremia in many countries including Thailand [2-4]. Infections caused by non-fermentative gram-negative bacteria (NFGNB) constitute an emerging problem in nosocomial setting, especially in an immunocompromised host. NFGNB are very problematic because of their ubiquitous distributions in the environment and their antimicrobial resistance patterns [5]. Data from the Surveillance and Control of Pathogens of Epidemiological importance (SCOPE) study revealed that approximately one-fourth of gram-negative bacteremia attributed to NFGNB [4].

Among all NFGNB, *Pseudomonas aeruginosa* and *Acinetobacter baumannii* are the most common causative pathogens for bacteremia [4,6,7]. Other uncommon NFGNB comprise many species such as *Stenotrophomonas maltophilia*, *Burkholderia* spp., *Alcaligenes* spp.*, Ralstonia* spp., *Sphingobacterium* spp., etc. [8]. Although many studies have investigated epidemiology of bacteremia caused by NFGNB, most studies focused only on *P. aeruginosa* and *A. baumannii*[4,9-11]. Knowledge of bacteremia caused by uncommon NFGNB is very limited. Lack of data on the disease epidemiology is a great obstacle to improve quality of care. Given these considerations, we conducted an observational study to explore the epidemiology of bacteremia caused by all groups of NFGNB.

## Methods

### Settings

This study was conducted at Siriraj hospital, a 2200-bed, tertiary-care university hospital in Bangkok, Thailand. The study protocol including waiver of informed consent for using patients' clinical isolates were approved by Siriraj Institutional Review board. From July 1, 2007 - December 31, 2008, all patients in whom at least one blood culture positive for NFGNB were prospectively identified through the microbiology laboratory database. Only patients who met the criteria of systemic inflammatory response syndrome (SIRS) within 24 hours before or after obtaining blood culture were enrolled in the study. If NFGNB were isolated on multiple occasions from the same patient, only the first episode of bacteremia was included.

### Microbiologic procedures and isolate identification

Microbiological and susceptibility results of all patients were retrieved from the microbiology laboratory database. All blood cultures during the study period were processed by the BactT/ALERT system (bioMeriéux) according to manufacturer’s protocol. Identification to the species level was achieved by using the Vitek 2 and/or API 20NE systems (bioMeriéux). All clinical isolates that could not be satisfactorily identified by the Vitek 2 or API 20NE were further characterized by 16S rDNA sequencing. Approximately 800 bp at the 5’ terminal of 16S rDNA gene was PCR amplified and sequenced using primers and protocols as described elsewhere [12,13]. The sequencing results were compared with databases in the GenBank (the nucleotide-nucleotide Basic Local Alignment Search Tool or BLAST).

Susceptibility testing was performed by Disk Diffusion or Broth Microdilution method as appropriate. Susceptibilities to each antimicrobial agent were determined according to criteria established by the Clinical and Laboratory Standards Institute [14,15]. Since the Clinical and Laboratory Standards Institute (CLSI) criteria for polymyxin E and tigecycline are not available, susceptibilities to tigecycline and polymyxin E were interpreted by using the European Committee on Antimicrobial Susceptibility Testing (EUCAST) clinical breakpoints [16]. We could not report the susceptibility results of some NFGNB species because the standard interpretation method is not available. Names of antimicrobial agent were abbreviated as follows: ceftazidime (CAZ), cefipime (FEP), ciprofloxacin (CIP), colistin or polymyxin E (CST), gentamycin (GEN), cotrimoxazole (SXT), imipenem-cilastatin (IMP), piperacillin-tazobactam (TZP) and tigecycline (TIG).

### Data collection

Medical records were retrospectively reviewed to obtain data including age, sex, hospital service, previous hospitalization, comorbidities, presence of a central venous catheter at the onset of infection, use of antimicrobial therapy or immunosuppressive agents in the preceding 30 days, suspected source(s) of bacteremia and infection-related mortality within 28 days after the onset of bacteremia (ID-mortality). The presence of the following comorbidities at the time of bacteremia was documented: cardiovascular diseases, chronic renal diseases, chronic liver diseases, chronic lung diseases, diabetes mellitus, neurological diseases, hematologic malignancy, solid cancer, HIV infection and receiving immunosuppressive therapy. We also recorded a number of blood cultures obtained, a number of positive blood cultures, other pathogens identified in the same set of blood cultures and hospital days both before and after the onset of bacteremia. We considered the date of obtaining the first positive blood culture as the onset of bacteremia.

### Definitions

*Pseudomonas aeruginosa* and *Acinetobacter baumannii* were considered the common NFGNB while all other NFGNB pathogens were considered the uncommon NFGNB.

Multidrug resistance was defined as resistance to carbapenems, second and third generation-cephalosporins, anti-pseudomonas penicillins, fluoroquinolones and aminoglycosides.

A bacteremic episode was considered to be hospital-acquired if one of the following criteria was true; 1) it occurred at least 48 hours after the admission and did not present or incubate at the time of admission; 2) it presented on the admission but the patient had been transferred from another medical center or long-term care facility, had spent at least 48 hours in the given facility; or 3) it presented on the admission but the patient has been hospitalized within the past 2 weeks [17].

A site of infection was determined by using the CDC definitions of nosocomial infections [17]. The site of infection was considered a suspected source of bacteremia if a similar NFGNB had also been isolated from that site within 24 hours before or after the onset of bacteremia. Therefore, each patient may have more than one suspected sources of bacteremia.

Receiving immunosuppressive agents was documented if the patient had a history of corticosteroid use (receipt of prednisone at a dosage of 20 mg per day (or equivalent) for at least 2 weeks) and/or history of receipt of chemotherapeutic agents in the preceding 30 days.

ID-mortality was defined as death in the setting of clinical evidence of active infection (elevated WBC and or elevated body temperature) and death within 5 days of last positive culture result.

### Statistical analysis

Descriptive statistics were used to express overall results. Univariate analysis was performed to determine an unadjusted association between the uncommon NFGNB bacteremia and other variables. Categorical variables were compared by using chi-square or Fisher’s exact test while continuous variables were compared by using t-test or Wilcoxon rank sum test as appropriate. To identify factors that independently associated with the uncommon NFGNB bacteremia, we subsequently built a multivariate logistic model by the stepwise method which is a combination of backward elimination and forward selection approaches. Variables were included in the multivariable model if they presented a p-value ≤0.20 in univariate analysis and then removed from the final multivariable model if they did not exhibit an adjusted p-value <0.05. A 2-tailed p-value of <0.05 was considered significant. All statistical calculations were performed by using STATA, version 12 (Stata Corp, College Station, TX).

## Results

### Distribution of causative pathogens

During the study period, there were a total of 221 patients with NFGNB bacteremia. Two patients (0.9%) had blood cultures positive for two species of NFGNB in a same set. The first patient had bacteremia caused by C*hryseobacterium meningosepticum* and *Elizabethkingia meningoseptica*. The second patient had bacteremia caused by *Ralstonia* spp. and *Burkholderia mallei*. Therefore, we detected 223 clinical isolates of NFGNB in 221 unique patients. In addition to a NFGNB pathogen, 25 patients (11.3%) also had a blood culture positive for a non-NFGNB pathogen, including *Escherichia coli* (n = 7), *Klebsiella pneumoniae* (n = 5), *Enterobacter* spp. (n = 3), *Enterococcus faecalis* (n = 2), *Staphylococcus aureus* (n = 2), alpha-hemolytic streptococci (n = 1) and fungi (n = 5). The most common combination was *P. aeruginosa* and *E. coli* (n = 7), followed by *P. aeruginosa* and *K. pneumoniae* (n = 5).

Approximately one-third of all clinical isolates (70/223) could not be identified by the standard biochemical methods. Of these 70 isolates, we could not perform the molecular study in 8 isolates due to an insufficient amount of specimens and we could not match the sequence of 11 isolates with our sequence database. A total of 51 isolates were successfully identified by the molecular method. The major causative pathogens were *Acinetobacter baumannii* (32.7%), followed by *Pseudomonas aeruginosa* (27.8%), *Stenotrophomonas maltophilia* (5.4%), *Acinetobacter lwoffii* (4.9%) and *Burkholderia pseudomallei* (2.7%). Distribution of causative pathogens and list of pathogen identified by the 16S rDNA sequencing are shown in Table 1.

**Table 1:** Distribution of causative pathogens

**Causative pathogen**	**Number of clinical isolates (%), n = 223**
**1. Pathogens identified by biochemical-based methods**	**153 (68.6)**
*Acinetobacter baumannii*	73 (32.7)
*Pseudomonas aeruginosa*	62 (27.8)
*Stenotrophomonas maltophilia*	12 (5.3)
*Burkholderia pseudomallei*	6 (2.7)
**2. Pathogens identified by 16S rDNA sequencing**	**51 (22.9)**
*Acinetobacter* spp. (other than *A. baumannii*)	11 (4.9)
*▪ Acinetobacter lwoffii*	10 (4.5)
*▪ Acinetobacter* spp. (unidentified species)	1 (0.4)
*Pseudomonas* spp. (other than *P. aeruginosa*)	12 (5.4)
*▪ Pseudomonas putida*	6 (2.7)
*▪ Pseudomonas stutzeri*	5 (2.2)
*▪ Pseudomonas* spp.	1 (0.4)
*Burkholderia* spp*.* (other than *B. pseudomallei*)	6 (2.7)
*▪ Burkholderia cepacia*	4 (1.8)
*▪ Burkholderia mallei*	1 (0.4)
*▪ Burkholderia* spp.	1 (0.4)
*Ralstonia* spp.	6 (2.7)
*▪ Ralstonia mannitolilytica*	2 (0.9)
*▪ Ralstonia pickettii*	1 (0.4)
*▪ Ralstonia* spp.	3 (0.9)
*Elizabethkingia meningoseptica*	2 (0.9)
*Chryseobacterium* spp.	2 (0.9)
*▪ Chryseobacterium menigosepticum*	1 (0.4)
*▪ Chryseobacterium* spp	1 (0.4)
*Acrobacter xylosoxidans*	2 (0.9)
*Aeromonas veronii biovar sobria*	1 (0.4)
*Agrobacterium* spp.	1 (0.4)
*Cupriavidus pauculus*	1 (0.4)
*Halomonas* spp.	1 (0.4)
*Herbassirillum huttiense*	1 (0.4)
*Roseomonas massiliae*	1 (0.4)
*Shewanella putrefaciens*	1 (0.4)
*Sphingomonas* spp.	1 (0.4)
*Wautersiella falsenii*	1 (0.4)
*Xanthomonas campestris*	1 (0.4)
3. Isolates that could not be identified by 16S rDNA sequencing*	**11 (4.9)**
4. Insufficient specimens**	**8 (3.6)**

**Note.** * The sequence of these isolates did not match our sequence database.

** Amount of these isolates was not enough for the molecular study.

### Susceptibility patterns

Details of antimicrobial susceptibility are shown in Table 2. Prevalence of multi-drug resistance (MDR) was 68.5% in *A. baumannii* and 5.1% in *P. aeruginosa.* Of the MDR isolates, CST was active against 87.2% of *A. baumannii* and 80% of *P. aeruginosa*. Less than 1/3 of *A. baumannii* isolates were susceptible to TIG. Despite the CST and TIG, the most active antibiotic was GEN (28.2%) for *A. baumannii,* TZP (91.5%) for *P. aeruginosa* and SXT (80.0%) for *S. maltophilia.* All of *B. pseudomallei* isolates (100.0%) were susceptible to CAZ and IMP while only 75% susceptible to SXT.

**Table 2:** Antimicrobial susceptibilities

**Antimicrobial agents**	**% Susceptible (number of susceptible isolates/number of tested isolates)**			
	***A. baumannii***	***P. aeruginosa***	***S. maltophilia***	***B. pseudomallei***
Ceftazidime	22.9 (16/70)	80.4 (45/56)	40.0 (2/5)	100.0 (6/6)
Cefepime	20.8 (15/72)	74.1 (40/54)	-	-
Piperacillin/tazobactam	20.6 (15/73)	91.5 (54/59)	-	-
Ciprofloxacin	18.6 (13/70)	75.4 (43/57)	-	-
Gentamycin	28.2 (20/71)	79.3 (46/58)	-	-
Imipenem/cilastatin	23.3 (17/73)	89.7 (52/58)	-	100.0 (6/6)
Cotrimoxazole	30.0 (21/70)	-	80.0 (8/10)	75.0 (45/60)
Multi-drug resistance*	68.5 (50/73)	5.1 (3/59)	-	-
Polymyxin E **	87.2 (41/47)	80.0 (4/5)	-	-
Tigecycline**	31.3 (10/32)	-	-	-

*% Resistant (number of resistant isolates/number of tested isolates).

** Polymyxin E and tigecycline were only tested against some multi-drug resistant isolates.

### Clinical characteristics

Detail of baseline characteristics, comorbidities and clinical features are shown in Table 3. Based on data from 221 unique patients, the median age [interquartile range] of our study subjects was 54 years [37–71] while the median length of hospital stay prior to the onset of bacteremia was 6 days [0–17]. Of these 221 patients, 116 (52.5%) were male and 167 (75.5%) had hospital-acquired bacteremia. Approximately 17% (9/54) of community-acquired bacteremia and 11% (18/167) of hospital-acquired bacteremia were polymicrobial. The three leading sources of bacteremia were pneumonia (34.4%), primary bacteremia (22.1%) and gastrointestinal tract (11.3%).

**Table 3:** Baseline characteristics, comorbidities and clinical features of patients in the uncommon NFGNB group vs. the common NFGNB group

**Variables**	**Uncommon NFGNB**	**Common NFGNB**	**Unadjusted OR**	**P-value**
	**(N = 86)**	**(N = 135)**	**[95% CI]**	
Median age (interquartile range), years	55 (37–70)	54 (37–71)	0.996 [0.984-1.007]	0.44
Median length of stay prior to bacteremia onset (interquartile range), days	1.5 (0–12)	10 (2–20)	0.996 [0.986-1.005]	0.37
Male sex	30 (34.9%)	86 (63.7%)	0.31 [0.17-0.56]	<0.001
**Services**				
▪ Medicine	49 (57.0%)	81 (60.0%)	0.54 [0.26-1.13]	0.13
*▪* Surgery	17 (19.8%)	36 (26.7%)	0.43 [0.18-1.00]	
*▪* Others	20 (23.2%)	18 (13.3%)	Reference group	
**Underlying diseases**				
*▪* Hematologic malignancies	11 (12.8%)	38 (28.2%)	0.37 [0.16-0.81]	0.007
*▪* Cardiovascular diseases	44 (51.2%)	45 (33.3%)	2.10 [1.16-3.78]	0.008
*▪* Chronic renal diseases	18 (20.9%)	17 (12.6%)	1.83 [0.83-4.06]	0.10
*▪* Chronic liver diseases	7 (8.1%)	9 (6.7%)	1.24 [0.38-3.91]	0.68
*▪* Chronic lung diseases	4 (4.7%)	9 (6.7%)	0.68 [0.15-2.55]	0.77*
*▪* Solid tumor	21 (24.4%)	27 (20.0%)	1.29 [0.64-2.59]	0.44
*▪* Diabetes	23 (26.7%)	26 (19.3%)	1.53 [0.76-3.05]	0.19
*▪* HIV infection	1 (1.2%)	1 (0.7%)	1.58 [0.20-124.65]	0.99*
*▪* Receiving immunosuppressive agents	6 (7.0%)	5 (3.7%)	1.95 [0.48-8.33]	0.28
*▪* Neurological diseases	2 (2.3)	6 (4.4%)	0.51 [0.05-2.96]	0.49*
**Recent antibiotic exposure within 30 d**				
*▪* All antibiotics	44 (51.2%)	95 (70.4%)	0.44 [0.24-0.80]	0.004
*▪* Beta-lactams	40 (46.5%)	88 (65.2%)	0.46 [0.26-0.84]	0.006
*▪* Cephalosporins	22 (25.6%)	53 (39.3%)	0.53 [0.28-1.00]	0.04
*▪* Carbapenems	13 (15.1%)	21 (15.6%)	0.97 [0.42-2.17]	0.93
*▪* Beta-lactam/beta-lactamase inhibitors	10 (11.6%)	24 (17.8%)	0.61 [0.25-1.42]	0.22
*▪* Fluoroquinolones	9 (10.5%)	12 (8.9%)	1.20 [0.42-3.26]	0.70
*▪* Aminoglycosides	5 (5.8%)	25 (18.5%)	0.27 [0.08-0.77]	0.007
*▪*Polymyxin E	3 (3.5%)	1 (0.7%)	4.84 [0.38-256.08]	0.30*
*▪* Vancomycin	13 (15.1%)	12 (8.9%)	1.83 [0.72-4.62]	0.15
*▪* Metronidazole	1 (1.2%)	15 (11.1%)	0.09 [0.002-0.64]	0.006*
*▪* Cotrimoxazole	0	6 (4.4%)	….	0.84*
*▪* Clindamycin	3 (3.5%)	9 (6.7%)	0.51 [0.09-2.11]	0.38*
**Clinical characteristics**				
Hospital-acquired infection	55 (64.0%)	112 (83.0%)	0.36 [0.18-0.72]	<0.001
Sites of infection				
• Primary bacteremia	31 (36.1%)	18 (13.3%)	3.66 [1.79-7.56]	<0.001
• Pneumonia	24 (27.9%)	52 (38.5%)	0.62 [0.33-1.15]	0.11
• Urinary tract	5 (5.8%)	15 (11.1%)	0.49 [0.14-1.51]	0.18
• Gastrointestinal tract	8 (9.3%)	17 (12.6%)	0.72 [0.25-1.85]	0.45
• Catheter-related infection	12 (13.9%)	9 (6.7%)	2.27 [0.83-6.39]	0.07
• Soft tissue and surgical site infection	0	7 (5.2%)	….	0.05*
• Febrile neutropenia	4 (4.7%)	15 (11.1%)	0.39 [0.09-1.29]	0.14*
• Others	1 (1.2%)	1 (0.7%)	1.58 [0.20-124.65]	0.99*
Infection-related mortality	15 (17.4%)	71 (52.6%)	0.19 [0.09-0.38]	<0.001

Note: * Fisher's exact p-value.

### Antimicrobial therapy

Due to a very high prevalence of multi-drug resistant pathogens, only half of patients (50.7%, 112/221) received at least one antimicrobial agent that active against causative pathogen(s) on the onset date. Percent of patients who received adequate antimicrobial therapy on the onset date was 66.1% in *P. aeruginosa*, 31.5% in *A. baumannii*, 16.7% in *S. maltophilia*, 100% in *B. pseudomallei* and 58.8% among the rest.

### Clinical outcomes

**Figure 1:**
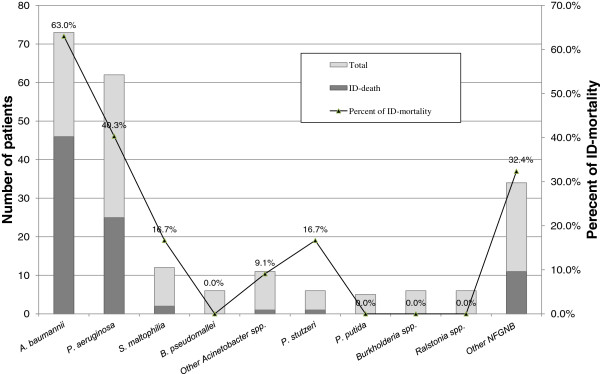
Number of total patients and patients who died due to infection (represented as a bar graph) and percent of ID-mortality (represented as a bar graph), classified by the causative pathogens.

The ID-mortality was 38.9% (86/221) in all study subjects, 52.5% (71/135) in the common NFGNB group and 17.4% (15/86) in the uncommon NFGNB group. The ID-mortality was highest among patients with *A. baumannii* bacteremia (63.0%, 46/73), followed *P. aeruginosa* bacteremia (40.3%, 25/52). There was no significant difference in the ID-mortality between those with monomicrobial bacteremia vs. polymicrobial bacteremia (39.7% vs. 33.3%; p = 0.53). Details of the ID-mortality across the causative pathogens are shown in Figure 1.

### Factors associated with uncommon NFGNB bacteremia

To identify factors associated with bacteremia caused by uncommon NFGNB, we subsequently compared characteristics of patients with uncommon NFGNB bacteremia (*S. maltophilia*, *B. pseudomallei* and all other uncommon NFGNB species) to patients with common NFGNB bacteremia (*P. aeruginosa* and *A. baumannii*). When baseline characteristics of patients in both groups were compared, a median length of hospital stay prior to the onset of bacteremia was slightly lower in the uncommon NFGNB group (1.5 day vs. 10 days). However, this difference did not reach statistical significance. Patients in the uncommon NFGNB group were less likely to have underlying hematologic malignancy as well as exposure to beta-lactams, aminoglycosides and metronidazole. However, the uncommon NFGNB group had a higher prevalence of underlying cardiovascular diseases.

The factors that were found to be independently associated with the uncommon NFGNB bacteremia are presented in Table 4. The independently associated factors included having primary bacteremia or catheter related blood stream infection as a source of bacteremia and previous exposure to vancomycin. Additionally, male sex, hospital-acquired infection and previous exposure to aminoglycoside were identified as protective factors in the multivariable model.

**Table 4:** Factors that independently associated with bacteremia caused by uncommon NFGNB (multivariable analysis)

**Uncommon NFGNB**	**Unadjusted OR [95% CI]**	**Adjusted OR [95% CI]**	**Multivariate**
			**P-value**
Male sex	0.31 [0.17-0.56]	0.28 [0.14-0.53]	<0.001
Previous exposure to aminoglycosides	0.27 [0.08-0.77]	0.23 [0.06-0.8]	0.01
Previous exposure to vancomycin	1.83 [0.72-4.62]	3.88 [1.35-11.1]	0.02
Hospital-acquired infection	0.36 [0.18-0.72]	0.23 [0.11-0.51]	<0.001
Primary bacteremia	3.66 [1.79-7.56]	6.43 [2.89-14.2]	<0.001
Catheter-related infection	2.27 [0.83-6.39]	4.48 [1.54-13.06]	<0.001

## Discussion

Our three leading causative pathogens were *A. baumannii*, followed by *P. aeruginosa* and *S. maltophilia.* These pathogens are commonly known as opportunists especially in the hospital setting. They are distributed ubiquitously in diverse environmental sources such as tap water or contaminated solutions [5]. Our distribution of causative pathogens was slightly different from previous studies that were conducted in the southern part of Thailand, US and Europe. All of these studies found that the three leading causative pathogens were *P. aeruginosa*, followed by *A. baumannii* and *S. maltophilia*[7,18-20]. We hypothesize that the remarkably high prevalence of *A. baumannii* may be the result of clonal spreading in our hospital. Unfortunately, a molecular study to confirm the clonal spreading was not performed at that moment.

Our fourth leading causative pathogen was *A. lwoffii* (formerly *A. calcoaceticus* var. *lwoffii*) which is well recognized as skin, oral and perineal flora [21]. Bacteremia caused by this pathogen is mostly related to catheter-related infection and has a good prognosis [21].

*B. pseudomallei*, the causative agent of melioidosis was noted as the fifth leading causative pathogen in our study. This pathogen is widely distributed in soil and rice paddies and considered an endemic pathogen in Southeast Asia especially Thailand.

Prevalence of multi-drug resistance among our *A. baumannii* isolates was considerably higher when comparing to other pathogens. Although several studies reported the excellent susceptibility of TIG against MDR-AB [22,23], but less than one-third of our multidrug-resistant *A. baumannii* (MDR-AB) isolates were susceptible to TIG. According to the susceptibility results, CST appeared to be the most optimal antimicrobial agent for treatment of MDR-AB-causing bacteremia in our hospital. Despite the high prevalence of MDR-AB, only 5% of *P. aeruginosa* isolates were multi-drug resistant; however, 80% of these MDR-PA were susceptible to CST.

CAZ and SXT given alone or in combination have been recommended as a treatment of choice for *S. maltophilia* infection [8]. According to our susceptibility result, however, SXT appeared to be the most promising therapeutic option.

Both CAZ and IMP showed 100% activity against *B. pseudomallei* while only 75% of *B. pseudomallei* were susceptible to SXT. Currently, CAZ- or IMP- based regimen is the preferable intravenous intensive-phase therapy while SXT is documented as the best oral eradication-phase therapy regardless the susceptibility result [24].

The ID-mortality in our study was quiet high, ranging from 17.4% in the uncommon NFGNB group to 63.0% in the *A. baumannii* group. The US surveillance study (SCOPE) reported only 34.0% crude mortality among patients with *A. baumannii* bacteremia [4] while another study conducted in Spain found that the ID-mortality among patients with NFGNB bacteremia was only 12.5% [7]. Nonetheless, both studies were conducted in the past decade which a prevalence of multidrug resistance was relatively low. We believe that the high ID-mortality was the result of the high prevalence of multidrug-resistance among *A. baumannii* pathogen. This hypothesis has been proved in previous publications [25,26].

In contrast to the common NFGNB, the ID-mortality in the uncommon NFGNB group was relatively low. Due to the high virulence of *A. baumannii* and *P. aeruginosa* infection, it would be very useful if physicians could distinguish patients who are at risk for *A. baumannii* and *P. aeruginosa* pathogens from those who are at risk for uncommon NFGNB. Given this reason, we performed additional analysis to identify factors associated with the uncommon NFGNB bacteremia. This would have important implications for selecting empiric antibiotic therapy when the identification and susceptibility results are not yet available. For the high risk patients, polymyxin E is probably the most promising choice in our institution.

Our study has several strengths compared with previous studies. First, while most studies focused only on *P. aeruginosa* and/or *A. baumannii*[9,20,27] we investigated all species of NFGNB. Second, our study included only patients with true bacteremia (patients who had at least one positive blood cultures for NFGNB and met the definition of SIRS within 24 hours of the onset of bacteremia), whereas other studies failed to distinguish true bacteremia from contamination [7]. Third, and most importantly, we also performed the molecular identification on all NFGNB isolates that could not be identified by conventional method. Without this, we would not be able to identify at least 20% of NFGNB isolates. We believed that our study provides thorough and accurate information on the distribution of causative pathogens.

Our study, however, has several potential limitations. First, many variables were obtained by chart-review. Therefore, informative bias and incompleteness of data may be issues. Additionally, some isolates could not be definitely identified to the species level by the 16S rDNA gene sequencing technique. This may be the result of the limitation of sequence database or sequence identity of some closely related species. Moreover, some clinical specimens were insufficient for molecular study. These may result in misclassification although it is unlikely this would result in differential bias.

## Conclusions

The epidemiology of NFGNB bacteremia in our hospital was slightly different from previous studies. Our study found the relatively higher ID-mortality among patients with *A. baumannii* and *P. aeruginosa* bacteremia. The independent factors associated with uncommon NFGNB bacteremia documented in this study can be used to distinguish the low risk patients from the high risk patients who would need empiric broad-spectrum antibiotics while waiting for species identification and susceptibility results.

## Competing interest

All authors report no potential conflict of interest.

## Authors’ contribution

PR was involved in study design, statistical analysis and writing the manuscript. PU was involved in data collection. PK and NA were involved in the conception of the study and writing the manuscript. All authors read and approved the final manuscript.

## Pre-publication history

The pre-publication history for this paper can be accessed here:

http://www.biomedcentral.com/1471-2334/13/167/prepub
